# Da-Huang-Fu-Zi-Tang Ameliorates Severe Acute Pancreatitis by Elevation of M2 Kupffer Cells in Rats

**DOI:** 10.1155/2021/5561216

**Published:** 2021-06-02

**Authors:** Yi Song, Yi Wang, Xin Qi, Xin Kang, Xiaoguang Lu

**Affiliations:** ^1^Department of Emergency Medicine, The Affiliated Zhongshan Hospital of Dalian University, Dalian 116001, China; ^2^Department of Emergency Intensive Care Unit, Yiwu Central Hospital, Yiwu 322000, China; ^3^Graduate School, Dalian University, Dalian 116622, China

## Abstract

**Introduction:**

Severe acute pancreatitis (SAP) is a clinical emergency often accompanied by inflammatory response syndrome (SIRS), which eventually leads to acute lung injury and failure of other organs. The activation of liver Kupffer cells (KCs) plays a major role in the process of SIRS and multiorgan damage caused by SAP. Da-Huang-Fu-Zi-Tang (DHFZT), a traditional Chinese prescription, has been widely used for SAP in the clinic. The present study investigated the activation state of KCs in SAP and the potential mechanism of DHFZT.

**Methods:**

A total of 24 Sprague Dawley rats were randomly assigned to four groups: SH (sham operation group + saline enema), SH-DHFZT (sham operation group + DHFZT enema), SAP (model group + saline enema), and SAP-DHFZT (model group + DHFZT enema). Blood samples were drawn from the abdominal aorta for measuring serum endotoxin, amylase, calcium ion, IL-1*β*, TNF-*α*, iNOS, and IL-10. Then, the pancreas, lung, liver, and ileum were harvested for histological observation, and the liver was used to detect the level of F4/80, CD86, and CD163 in KCs with immunohistochemistry and western blot.

**Results:**

In the SAP group, the CD86+ KCs were significantly increased with a high level of IL-1*β*, TNF-*α*, and iNOS, and the organs were impaired. In the SAP-DHFZT group, CD163+ KCs were significantly increased with the high level of IL-10, and the damage to organs was alleviated.

**Conclusion:**

These phenomena suggested that the SIRS and multiple organ damage in SAP might be related to the excessive activation of M1 KCs, and DHFZT might alleviate the SIRS by inducing the differentiation of KCs into the M2-type and promote the expression of anti-inflammatory factor IL-10.

## 1. Introduction

Systemic inflammatory response syndrome (SIRS) caused by severe acute pancreatitis (SAP) often leads to serious complications, such as acute respiratory distress syndrome (ARDS) and multiple organ dysfunction syndromes (MODS); the mortality rate is up to 30% [1]. It was speculated that the infiltration and metastasis of inflammatory factors released by local inflammation of the pancreas were the main sources of inflammatory substances in vivo. However, gradually, the release of local inflammatory mediators in pancreatitis was detected, and the inflammatory factors released by the excessive defense of subsequently activated macrophages, lymphocytes, and other immune cells were found to be the real culprits of uncontrolled development of SAP [2]. Also, studies have shown that macrophages are critical for this process [2, 3] and, hence, are the focus of research on the pathogenesis of SAP.

In recent years, the Kupffer cells (KCs), which reside in the liver, account for 80–90% of all human tissue macrophages. Relying on pattern recognition receptors (PRR), the KCs can differentiate into M1 and M2 two different types under different stimulations [4, 5]. M1-type belongs to the classical activation of macrophages [6], which is induced by interferon-*γ* (IFN-*γ*) alone or together with the microbial products (for example, lipopolysaccharide (LPS)) and inflammatory cytokines (tumor necrosis factor (TNF)). CD86, specifically expressed in M1 KCs, is the main cofactor of inducing T lymphocyte proliferation and producing IL-2. These CD86+ KCs have high antigen expression ability and produce a large number of proinflammatory factors, such as IL-1, IL-12, IRF-5, and nitric oxide (NO), which exert antimicrobial activity in the process of bacterial infection [7, 8]. In the 1990s, Stein and Doyle reported another activated macrophage phenotype M2 induced by IL-4 and IL-13 [9, 10]. Compared to the M1-type, the M2-type can specifically express CD163. The CD163+ KCs have the characteristic high secretion of IL-10 but the low secretion of IL-12 and other proinflammatory factors (IL-1, TNF, and IL-6) [9–11]. The IL-10 secreted by M2 KCs can inhibit T cells and participate in the process of immune tolerance [11], which is essential to prevent an excessive immune response. Together, M1 and M2 KCs play a critical role in the regulation of the body's inflammatory response that affects the development and outcome of the disease. Some researchers found that M2 macrophages continue to replace M1 during the recovery stage in typhoid patients until the inflammation regressed completely [12]. Also, the activation of KCs is crucial for the development of liver fibrosis, viral hepatitis, and even liver cancer [13–15]; however, the specific pathway and mechanism are yet explored. In addition, studies found that applying gadolinium chloride blocks the function of KCs in SAP rats, which reduces the serum cytokine level and eventually alleviates acute lung injury [16–18]. In SAP, intestinal mucosal injury leads to the translocation of bacteria and endotoxin. The liver is the only method of portal blood carrying bacteria and endotoxin; moreover, the blood flow in hepatic sinuses is slow, which creates a convenient condition for KCs to interact with these endogenous or exogenous substances. Therefore, we speculated the pivotal role of KCs in the inflammatory response of SAP.

Da-Huang-Fu-Zi-Tang (DHFZT) is a famous traditional Chinese prescription that has been widely used to treat various inflammatory diseases, and our preliminary study demonstrated that DHFZT enema alleviates lung injury with SAP [19]. Patients with SAP are often given fasting water. Also, gastrointestinal decompression is continued in the early stage, rendering DHFZT enema an effective clinical treatment for SAP patients [19, 20]. In addition, the previous basic research showed that DHFZT protects intestinal mucosa and reduces the release of inflammatory cytokines in SAP [21]. DHFZT exerts an immunopharmacological effect similar to that of VGX-1027 [22, 23], both of which reduce the secretion of proinflammatory cytokines produced by macrophages like IL-1, TNF-*α*, and IL-10 in vitro stimulated by lipopolysaccharide; however, the mechanism underlying the treatment of SAP is not yet fully elucidated. Thus, the present study investigated the activation of KCs and their significance in SAP rats, thereby providing some theoretical basis for the treatment of SAP with traditional Chinese medicine DHFZT.

## 2. Materials and Methods

### 2.1. Animals

A total of 24 Sprague Dawley (SD) male rats, weighing 220–280 g, were purchased from the Experimental Animal Center of Dalian Medical University. The animals were randomly assigned to four groups (*n* = 6): SH (sham operation + saline enema), SH-DHFZT (sham operation + DHFZT enema), SAP (model + saline enema), and SAP-DHFZT (model + DHFZT enema). Cages were individually ventilated at 22 ± 2°C and 45–65% relative humidity with a photoperiod of 12 h light and 12 h dark. All procedures were in compliance with the National Institute of Health Guide for the Care and Use of Laboratory Animals and were approved by the Animal Research Ethics Committee of Affiliated Zhongshan Hospital of Dalian University. Anesthetic drugs and all other necessary measures were used to reduce animal suffering during experimental procedures.

### 2.2. Preparation of DHFZT

DHFZT was prepared, as described previously [21], and the formula of DHFZT is shown in Table 1. To keep the consistency of the herbal chemical ingredients, all of the herbal components were originally obtained from the standard native sources (Tong Ren Tang Group Co., Ltd., Beijing, China), and the decocting and quality control of the drugs were completed by the Pharmacy Department of Zhongshan Hospital Affiliated to Dalian University. The main chemical compositions of DHFZT have been analyzed in our previous study [21]. After decocting and disinfecting in a 100°C water bath, the drugs were stored at 4°C. Before the enema, the drugs were heated to 37°C and agitated.

### 2.3. Experimental Process

All rats were adaptively fed for 1 week before the experiment. Then, the rats fasted for 8 h, with water deprivation for 4 h before the operation. Anesthesia was induced with inhalation of 5% isoflurane and maintained with 2% isoflurane. The abdominal cavity was exposed aseptically through a median abdominal incision. For the SAP and DHFZT groups, the liver was lifted with a cotton swab moistened with normal saline to disclose the pancreas and pancreaticobiliary duct, and then the pancreaticobiliary duct was ligated at the portal end of the liver. At the opening near the duodenal papilla, 4% sodium taurocholate (1 mL/kg) was injected into the pancreaticobiliary duct by retrograde puncture with a No. 1 needle. For the SH and SH-DHFZT groups, the pancreas was exposed similarly, and then, the abdomen was closed. The SH/SAP group was administered 2 mL normal saline enema at 12, 24, and 36 h after the operation; the SH-DHFZT/SAP-DHFZT group was given 2 mL DHFZT enema at the same time point. At 48 h after the operation, 5 mL blood samples were withdrawn from the abdominal aorta for measuring serum endotoxin, amylase, calcium ion, IL-1*β*, TNF-*α*, iNOS, and IL-10. Subsequently, the pancreas, lung, liver, and ileum were harvested for histological observation; the liver tissue was also used for detection of the expression of F4/80, CD86, and CD163 in KCs with immunohistochemistry (IHC) and western blot.

### 2.4. Estimation of Serum Endotoxin, Amylase, and Calcium Ion

The supernatant from the blood samples was collected by centrifugation at 3000 rpm and 4°C for 5 min. This supernatant was used for endotoxin detection by MB-80 microbial fast dynamic monitoring system (Jinshanchuan Technology Development Co., Ltd, Beijing, China), as well as for amylase and calcium ion detection by an automatic biochemical analyzer (Siemens, German).

### 2.5. Enzyme-Linked Immunosorbent Assay for IL-10, TNF-*α*, iNOS, and IL-1*β*

The supernatant from the blood samples was collected by centrifugation at 1,000 rpm and 4°C for 15 min and then stored at −80°C. The commercial enzyme-linked immunosorbent assay kits (Westang Biotech Co., Ltd, Shanghai, China) were used for the quantitative measurement of serum IL-10, iNOS, TNF-*α*, and IL-1*β* in each group, according to the manufacturer's instructions.

### 2.6. IHC and Histological Detection

IHC staining was performed using the DAB chromogenic detection kit (Zhongshan Jinqiao Technology Co., Ltd, Beijing, China) according to the manufacturer's instructions. The following antibodies were utilized: rat anti-CD163 polyclonal antibody, rat anti-CD86 polyclonal antibody, and rat anti-F4/80 polyclonal antibody (Abcam, Shanghai, China). Paraffin sections of the collected tissues were routinely stained with hematoxylin-eosin (HE) for histological observation. After IHC and HE staining, the images were captured by a microscope (Nikon Co., Japan) and recorded using ISCapture imaging software.

### 2.7. Western Blot

Liver tissues were prepared as described above. Proteins were extracted using Nucleus and Cytoplasm Protein Extraction Kit (Solarbio Technology Co., Ltd, Beijing, China), according to the manufacturer's instructions. The concentration of the total proteins in each group was determined using the BCA Protein Assay Kit (Solarbio Technology Co). Western blot analysis was performed as described previously [24]. The primary antibodies used for the analysis included rat anti-CD163 polyclonal antibody, rat anti-CD86 polyclonal antibody, and rat anti-F4/80 polyclonal antibody (Abcam). Horseradish peroxidase-conjugated antibodies against rabbits or mice (Santa Cruz Biotechnology, Santa Cruz, CA, USA) were used as secondary antibodies. The immunoreactivity of the protein bands was visualized with a chemiluminescence detection system using the SuperSignal substrate.

### 2.8. Statistical Analysis

Data are presented as the mean ± SD. All statistical analyses were performed using the GraphPad (version 8.0). The data were evaluated for statistical significance with a one-way analysis of variance (ANOVA) followed by Tukey's test. *P* < 0.05 was considered statistically significant.

## 3. Results

### 3.1. SAP Caused Obvious Pathological Changes in Rats

**Figure 1:**
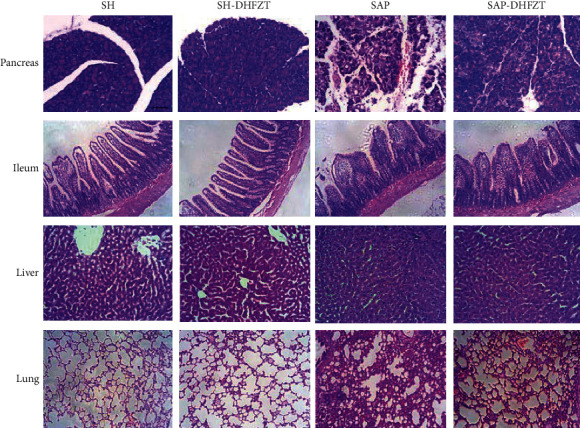
Histological observation of the pancreas, ileum, liver, and lung tissue with hematoxylin-eosin staining. The structure of the pancreas, ileum, liver, and lung was intact in the sham and sham-DHFZT groups, but these tissues were damaged in the SAP group. The tissue damage was alleviated in the SAP-DHFZT group. SH: sham; DHFZT: Da-Huang-Fu-Zi-Tang; SAP: severe acute pancreatitis. Scale bar = 100 *μ*m.

The serum amylase and endotoxin levels in the SAP group were significantly higher than those in the SH group, while the serum calcium ion level was significantly decreased (Table 2). Moreover, the structure of the pancreas, ileum, liver, and lung was intact in the SH group but was obviously damaged in the SAP group, with large numbers of infiltrated inflammatory cells in the pancreatic and lung tissues (Figure 1).

### 3.2. SIRS and Activation of KCs Might Play a Major Role in the Pathogenesis of SAP

**Figure 2:**
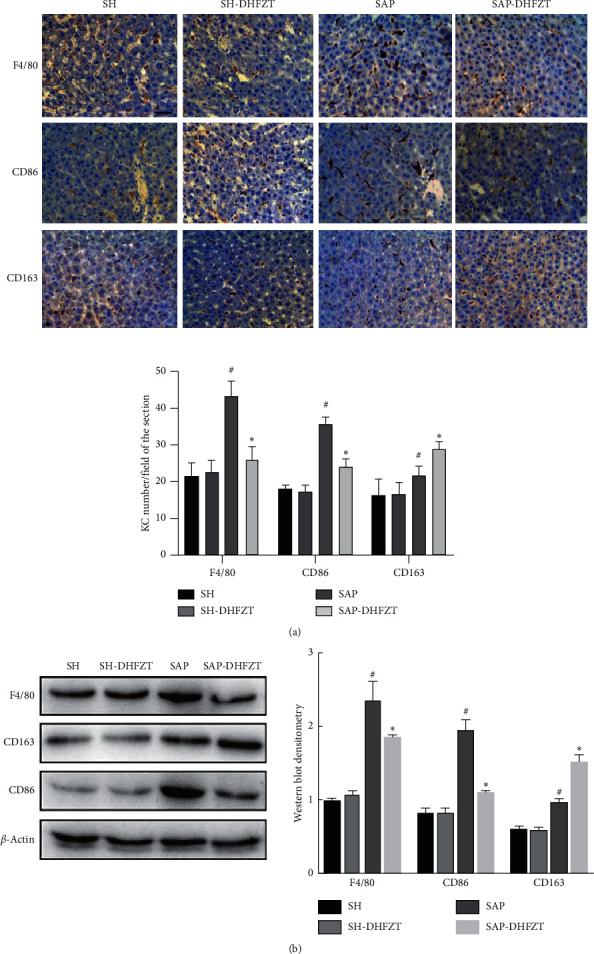
The expression of F4/80, CD86, and CD163 in the liver tissue. (a) IHC and statistical analysis of F4/80, CD86, and CD163 were performed in each group. (b) Protein expression levels and western blot analysis of F4/80, CD86, and CD163 in each group. ^#^*P* < 0.05, the SAP group versus the SH group; ^*∗*^*P* < 0.05, the SAP-DHFZT group versus the SAP group; SH: sham; DHFZT: Da-Huang-Fu-Zi-Tang; SAP: severe acute pancreatitis. Scale bar = 100 *μ*m.

We measured the level of IL-10, TNF-*α*, iNOS, and IL-1*β* to investigate the inflammatory response in SAP. The levels of these molecules were significantly higher in the SAP group than those in the SH group (Table 3). Moreover, the IHC and western blot analysis showed that the expression of F4/80, CD86, and CD163 in the KCs of the SAP group was significantly increased as compared to that in the SH group (Figures 2(a) and 2(b)).

### 3.3. Pathological Condition of SAP Could Be Improved under the Intervention of DHFZT

Compared to the SAP group, the amylase and endotoxin levels were obviously decreased, but the calcium ion level was significantly increased in the SAP-DHFZT group (Table 2). In addition, the damage to tissues was significantly mitigated with decreased infiltration of inflammatory cells in the SAP-DHFZT group (Figure 1).

### 3.4. DHFZT Improved SAP by Regulating KCs and Alleviating SIRS

Compared to the SAP group, the levels of serum iNOS, IL-1*β*, and TNF-*α* were decreased markedly, while that of IL-10 was increased significantly in the SAP-DHFZT group (Table 3). The expression of F4/80 and CD86 in KCs was obviously decreased, but that of CD163 was significantly increased in the SAP-DHFZT group (Figure 2(b)). Correspondingly, the number of CD86+ KCs was significantly decreased, while that of CD163+ KCs was significantly increased in the SAP-DHFZT group (Figure 2(a)).

## 4. Discussion

In SAP, the body successively suffers from a local pancreatic injury, external organ damage, and systemic inflammatory response, and hence, the “three-hit theory” has been widely recognized. Mayerle and Mikami [2, 3] proposed that the systemic inflammatory response was the strongest hit, following which the inflammatory factors released in bursts destroyed the balance of the internal environment, further aggravating the damage of vital organs and causing the vicious circle of disease. The liver is a popular place for intestinal bacteria and their products. The changes caused by a large number of intestinal bacteria and endotoxin entering the liver play a major role in the progress of SAP. Some studies showed that the KCs, which recognized LPS by toll-like receptor (TLR), could be activated to maintain immune activation and immune tolerance and help the body resist foreign bacterial infection [25, 26]. Immune activation and immune tolerance are mainly accomplished by two types of KCs with similar morphology but different functions; i.e., M1 plays a proinflammatory role, and M2 promotes tissue repair and helps the body to effectuate immune tolerance.

The current results showed that in SAP, the intestinal villi were obviously damaged due to the massive aggregation of the inflammatory cells and erythrocytes, and consequently, the serum endotoxin level was increased. Also, the intestinal epithelial cells observed by scanning electron microscopy in our previous study had shown that the membrane of the intestinal mucosal epithelial cell was broken; the cell contour was ambiguous; the cell arrangement was disordered; the groove of corrugation disappeared in SAP rats [21]. Under this condition, the massive bacteria and their products, which break through the impaired intestinal barrier and are carried to the liver by the portal vein, will bring unprecedented impact to the KCs and generate hypersensitivity reactions. Reportedly, the expression or sensitivity of TLR4 on activated KC surface would increase with prolonged exposure to LPS [27]. The receptors on the surface of KCs interact with LPS to trigger the synthesis of inflammatory mediators, such as IL-6, IL-12, IL-1*β*, TNF-*α*, and NO. These inflammatory mediators can inhibit the proliferation of microorganisms while damaging the body due to the oxidative stress response [28, 29]. In this study, we found that, compared to the sham group, the number of F4/80-labeled KCs in the liver of the SAP group increased significantly; both CD86-labeled M1 KCs and CD163-labeled M2 KCs were also increased markedly. In addition, the high levels of serum iNOS, TNF-*α*, and IL-1*β* secreted by M1 KCs were detected, accompanied by obvious pulmonary edema, pancreatic necrosis, and intestinal mucosal damage in SAP rats. These findings suggested that the activated M1 KCs might play a leading role in SIRS and organ injury following SAP. Furthermore, the serum level of IL-10 in SAP rats was only slightly increased, which suggested that the activated KCs differentiate into M1-type to defend intestinal bacteria and endotoxin in the early stage of SAP. The lack of M2 KCs and the anti-inflammatory factor IL-10 and the proinflammatory response mediated by M1 KCs are currently predominant features in SAP. However, this one-sided differentiation leads to a breakdown of the balance between proinflammatory and anti-inflammatory responses, which might be the main cause of SIRS and MODS in SAP.

After treatment with DHFZT, the expression of F4/80 and CD86 was significantly attenuated, and the serum levels of TNF-*α*, iNOS, and IL-1*β* were also significantly decreased, indicating that the activation of M1 KCs was reduced. Moreover, the expression of CD163 and IL-10 was increased significantly, accompanied by the alleviation of lung injury, pancreatic necrosis, and intestinal mucosal damage. Furthermore, IL-10 inhibits the production of LPS-stimulated inflammatory cytokines, such as IL-1*β*, IL-6, IL-8, and TNF-*α*, by inhibiting the transcription of genes with rapid onset, albeit in a dose-dependent manner [30]. Combined with the results of this study, we speculated that DHFZT could reverse the one-sided activation of KCs to the M1-type and promote the activation of M2 KCs in SAP. Then, the increased secretion of anti-inflammatory factor IL-10 can regulate the balance of proinflammatory and anti-inflammatory responses to mitigate SIRS and organ damage. Reportedly, the upregulation of IL-10 in the model of pancreatitis, along with the inhibition of TNF-*α*, was induced by heme oxygenase-1 (HO1) [31]. Some agents, such as luteolin and curcumin, improved the SAP in rodents by induction of HO1 [32, 33]. Nonetheless, future studies are required to confirm whether DHFZT could improve SAP by inducing HO1 and its possible association with the anti-inflammatory Nrf2/HO-1 signaling pathway, in order to reveal the immunopharmacological mechanism of DHFZT in SAP.

## 5. Conclusions

The present study suggested that the SIRS in SAP may be related to the excessive activation of M1-type KCs. DHFZT may alleviate the SIRS in rats with SAP by inducing the differentiation of KCs into M2-type and promoting the expression of anti-inflammatory factor IL-10. Additional studies will be necessary for identifying if DHFZT or its main compounds can promote the KCs to differentiate into M2-type KCs in vitro.

## Figures and Tables

**Table 1:** Herbal compositions of DHFZT.

Scientific name	Herbal name	Quantity (dry, g)
*Rheum palmatum* Linn.	Radix et Rhizoma Rhei (DH)	9.0
*Aconitum carmichaelii*	Debeaux Radix Aconiti Lateralis Praeparata (FZ)	9.0
*Asarum heterotropoides* F. Schmidt var. *mandshuricum*	Radix et Rhizoma Asari (XX)	3.0
Total		21.0

DHFZT = Da-Huang-Fu-Zi-Tang. DH = Da Huang. FZ = Fu Zi. XX = Xi Xin.

**Table 2:** Serum endotoxin, amylase, and calcium ion content in each group.

Groups	Endotoxin (pg/L, *n* = 6)	Amylase (U/L, *n* = 6)	Calcium ion (mmol/L, *n* = 6)
SH	37.11 ± 4.34	192.83 ± 4.25	2.11 ± 0.04
SH-DHFZT	38.75 ± 1.71	193.22 ± 5.01	2.02 ± 0.06
SAP	163.0 ± 4.02^##^	1429.37 ± 36.37^#^	1.37 ± 0.06^#^
SAP-DHFZT	83.66 ± 2.47^∗^	600.1 ± 27.49^∗^	1.84 ± 0.03^∗^

Values are mean ± SD. ^#^*P* < 0.05, the SAP group versus the SH group. ^*∗*^*P* < 0.05, the SAP-DHFZT group versus the SAP group. SH = sham. DHFZT = Da-Huang-Fu-Zi-Tang. SAP = severe acute pancreatitis.

**Table 3:** Serum IL-1*β*, TNF-*α*, IL-10, and iNOS level in each group.

Groups	IL-1*β* (pg/mL, *n* = 6)	IL-10 (pg/ml, *n* = 6)	iNOs (ng/m, *n* = 6)	TNF-*α* (pg/ml, *n* = 6)
SH	24.93 ± 1.12	36.14 ± 1.82	52.11 ± 5.27	20.81 ± 0.19
SH-DHFZT	24.87 ± 0.95	35.02 ± 1.86	51.42 ± 5.42	20.71 ± 0.17
SAP	88.01 ± 1.22^#^	43.17 ± 0.92^#^	108.25 ± 7.24^#^	60.36 ± 2.11^#^
SAP-DHFZT	66.73 ± 3.03^∗^	88.03 ± 3.21^∗^	65.14 ± 5.59^∗^	43.23 ± 1.79^∗^

Values are mean ± SD. ^#^*P* < 0.05, the SAP group versus the SH group. ^*∗*^*P* < 0.05, the SAP-DHFZT group versus the SAP group. SH = sham. DHFZT = Da-Huang-Fu-Zi-Tang. SAP = severe acute pancreatitis.

## Data Availability

All the data were acquired from the Center Lab of the Affiliated Zhongshan Hospital of Dalian University.
